# Supplementary material for: Magnetic vector tomography reveals giant magnetofossils are optimised for magnetointensity reception
Source: Commun Earth Environ. 2025 Oct 20;6(1):810. doi: 10.1038/s43247-025-02721-3 (PMC12537488; doi:10.1038/s43247-025-02721-3)
Supplement: Supplementary file 2 — Supplemental Material [file 43247_2025_2721_MOESM2_ESM.pdf]

Magnetic vector tomography reveals giant magnetofossils are optimised for magnetointensity reception

Supplemental Information

Supplementary Methods

1. Torque-transducer model

For details of the Torque-transducer model used to calculate Fig. 4, refer to the original paper by Winklhofer and Kirschvink (2010). Fig. 4b is calculated using their Equations C4 and D3b and is equivalent to their Fig. 2:

$$\Delta\psi = \langle\psi^2\rangle - \langle\psi\rangle^2 \qquad \text{Equation S1}$$

$$\langle\psi^2\rangle(\theta = \pi) = 3\left[1 - \frac{K}{MB} + \sqrt{\left(1 - \frac{K}{MB}\right)^2 + \frac{4k_BT}{6MB}}\right] \qquad \text{Equation S2}$$

where $\langle\psi\rangle = 0$ for $\theta = 180°$, K is the tortional stiffness of the anchoring pivot, M is the remanent and/or induced moment of the particle, B is the field strength, k_B is Boltzmann's constant, and T is temperature. The inset to Fig. 4b is the numerical first derivative of Equation S1 with respect to (MB/K). Fig. 4c (solid circles) shows the value of this derivative calculated at the critical point ($MB/K = 1$) as a function of K. The minimal detectable angle of deviation from perfect antiparallel alignment with the field (η, open circles in Fig. 4c) was calculated at the critical point using Equation D9 from Winklhofer and Kirschvink (2010):

$$\eta = \frac{k_BT}{MB}\frac{1}{\Delta\psi(\theta=180°)} \qquad \text{Equation S3}$$

where $\Delta\psi$ is given by Equation S1.

2. Susceptibility of the giant spearhead versus magnetotactic bacteria

The contribution of anisotropic susceptibility to the magnetic torque (D) exerted on the particle by the Earth's field was calculated using Equation 2.2 of Winklhofer and Kirschvink (2010):

$$\boldsymbol{D} = \mu_0 V\,(\chi\boldsymbol{H}) \times \boldsymbol{H} \qquad \text{Equation S4}$$

where V is volume, χ is the susceptibility tensor (in SI units), H is the magnetic field. The anisotropy of magnetic susceptibility was determined micromagnetically using a model with a circular cross section. Starting from the GEM state with no Bloch points, the slope of the magnetisation components, M_x, M_y and M_z with respect to fields applied in the x, y and z directions were determined by least squares fitting for fields between -1 mT and + 1 mT:

$$\chi_{ij} = \begin{pmatrix} \frac{dM_x}{dH_x} & \frac{dM_x}{dH_y} & \frac{dM_x}{dH_z} \\ \frac{dM_y}{dH_x} & \frac{dM_y}{dH_y} & \frac{dM_y}{dH_z} \\ \frac{dM_z}{dH_x} & \frac{dM_z}{dH_y} & \frac{dM_z}{dH_z} \end{pmatrix} = \begin{pmatrix} 2.6 & 0.0031 & -0.213 \\ 0.0019 & 2.582 & 0.0021 \\ -0.208 & 0.00384 & 6.48 \end{pmatrix} \qquad \text{Equation S5}$$

Combining Equations S4 and S5, the maximum torque experienced by the giant magnetofossil due to induced moments is 4.5 x 10^{-21} Nm at θ = 42°. This compares with the maximum torque due to the remanent moment of 5 x 10^{-19} Nm at θ = 90°. Hence, the maximum torque due to anisotropic induced magnetisation is of the order 1% of the maximum torque created by the remanent magnetisation of the spearhead, so is likely of only secondary importance.

For comparison, the magnetic susceptibility of six conventional magnetotactic bacteria chain models with cuboctahedral, prismatic, and bullet-shaped morphologies taken from Pei et al. (2023) were calculated using MERRILL under applied fields parallel to the chain axis, ranging from -1 mT to 1 mT with a step of 0.05 mT (Table S2).

Supplementary Figures and Tables

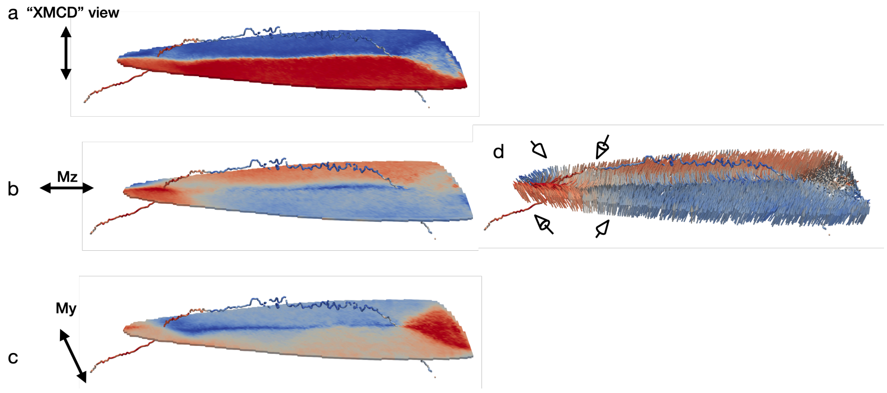

Figure S1. Longitudinal slices through the 3D magnetic vector tomography reconstruction of the giant spearhead magnetofossil (see also Supplemental Movie S2). Panels on the left show (a) M_x (i.e., the component of magnetisation imaged by phase XMCD), (b) M_z (i.e., the component parallel to the length of the particle) and (c) M_y. (d) highlights the tilting of moments away from the basal plane towards the ± z directions. Note the reversal of M_z components in the tip versus body of the particle. The trajectory of the vortex core is shown for reference.

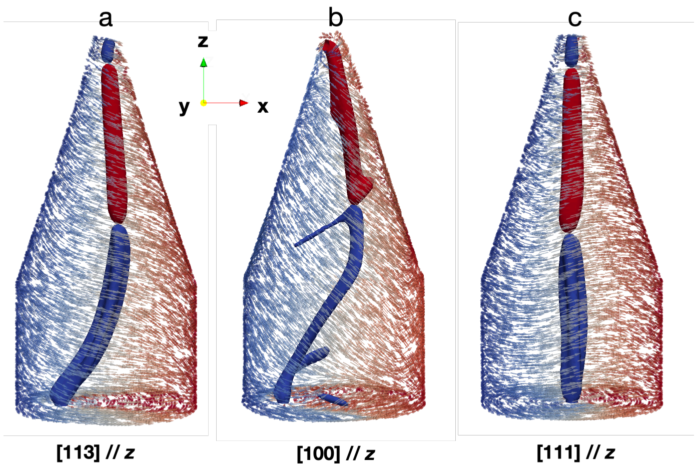

Figure S2. Micromagnetic simulations performed using a simplified model (cylinder + cone) and a circular cross section (r_x = 0.55 µm) with a range of crystallographic orientations: (a) [113] parallel to z, (b) [100] parallel to z, and (c) [111] parallel to z. Surface spins are coloured according to the

component of magnetisation normal to the plane of the diagram. The vortex core is shown using a contour surface of $\boldsymbol{M} \cdot (\nabla \times \boldsymbol{M})$ (red positive, blue negative). Pinch points along the core correspond to Bloch points. A [113] orientation provides the best agreement with experimental observations. Best agreement with observations is obtained with a [113] orientation. This orientation places the nearest <111> magnetocrystalline easy axis at an angle of 29.5° to the dominant shape anisotropy easy axis (z) of the particle. The non-parallel easy axes define the medial plane that hosts both the curved vortex core trajectory and the medial domain wall, with the core orientation being dominated by magnetocrystalline anisotropy in the base and by shape anisotropy in the cone. As observed, the core trajectory is predicted to initiate at the base of the particle, near an outer edge, and terminates close the tip. The exact core trajectory is sensitive to the precise shape and sharpness of the tip, as well as the applied field history.

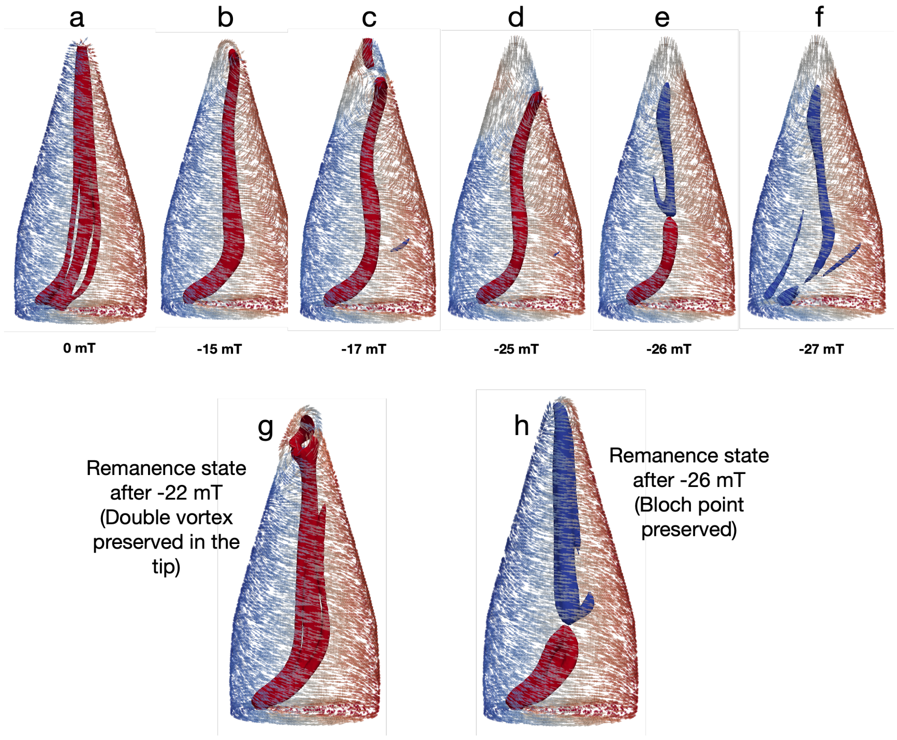

Figure S3. Micromagnetic simulations performed with an elliptical cross section (r_x = 0.55 µm; r_y = 0.4675 µm), [113] parallel to z and [111] (the magnetocrystalline easy axis) in the x-z plane for a range of magnetic fields applied along z, as indicated in (a-f). The starting configuration for all simulations is the GEM states shown in (a). Surface spins are coloured according to the component of magnetisation normal to the plane of the diagram. The vortex core is shown using a contour surface of $\boldsymbol{M} \cdot (\nabla \times \boldsymbol{M})$ (red positive, blue negative). Pinch points along the core correspond to Bloch points. (g) Remanent state obtained after applying a field of -22 mT along z. A reversal of vortex helicity is preserved in the tip of the particle, but no Bloch point. (h) Remanent state obtained after applying a field of -26 mT along z. The Bloch point seen in (e) is preserved at remanence.

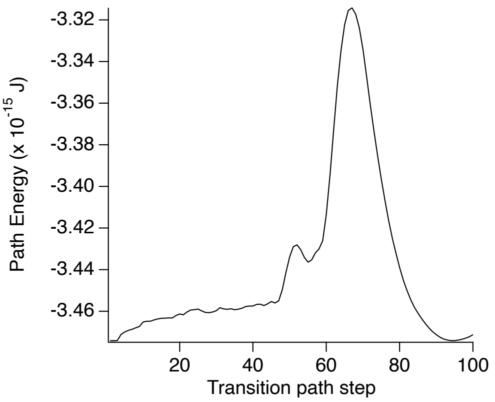

Figure S4. Minimum action path energy as function of path step corresponding to the nucleation (left), propagation (centre) and annihilation (right) of the Bloch point seen in Supplemental Movie S4. The minimum energy required to nucleate a Bloch point at the tip is 700 k_BT, correponding to a relaxation time greater than the age of the universe. The large peak to the right corresponds to the destruction of the Bloch point at the base.

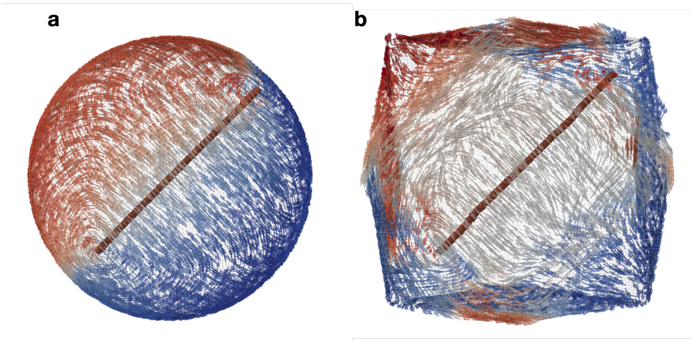

Figure S5. Lowest energy remanence states of (a) sphere and (b) cuboctahedron with equal volume to the giant magnetofossil (1.15 x 10^{-18} m^3; equivalent sphere diameter 1.3 μm). The remanence in both cases is a {111} easy axis aligned single vortex. Remanent moments of these particles are 2.96 x 10^{-15} Am^2 and 6.09 x 10^{-15} Am^2, respectively, factors of 1.64-3.4 smaller than the moment the giant magnetofossil. Both the larger moment and lack of multiple easy axes are advantageous from a magnetoreception perspective.

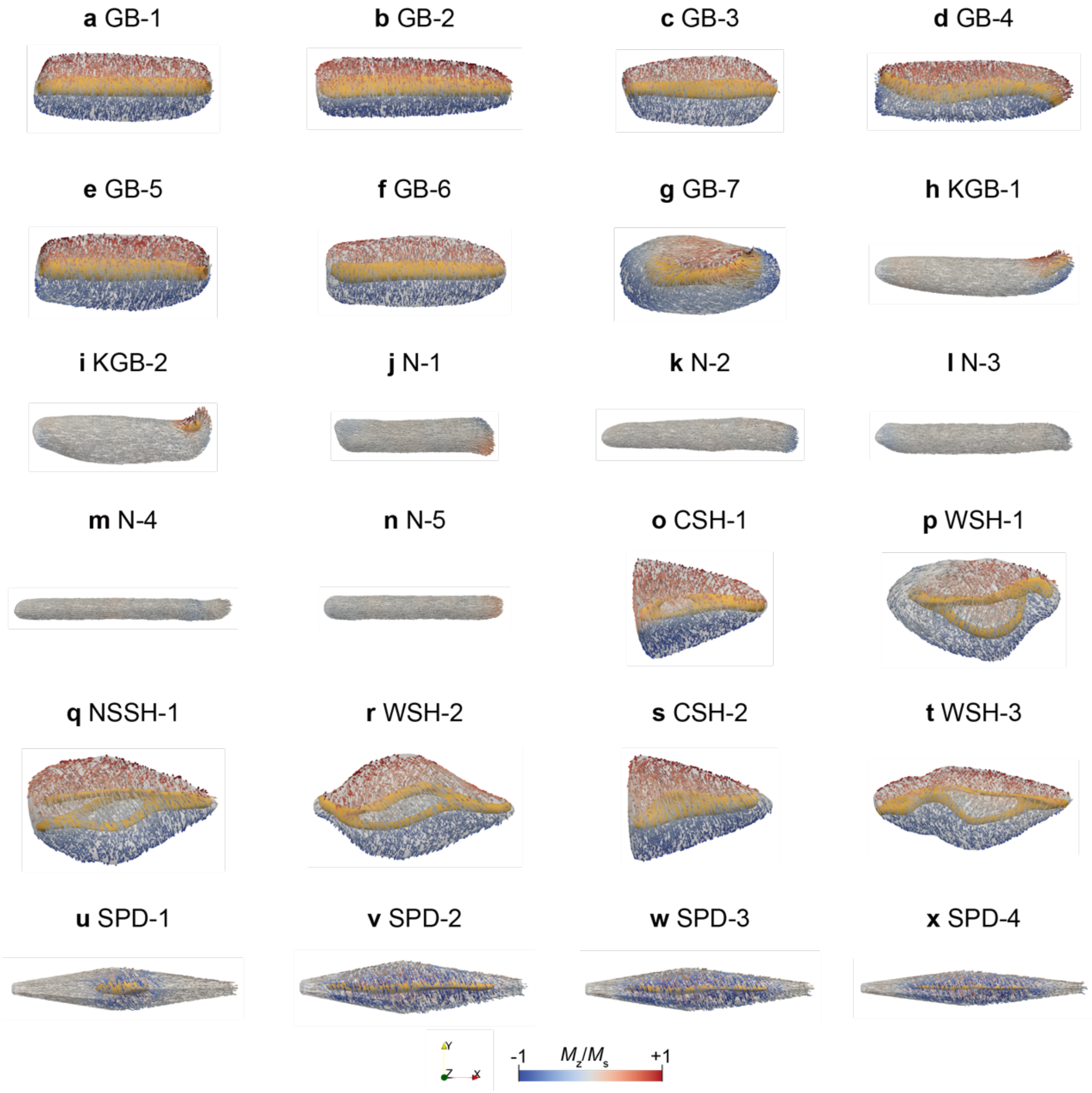

Figure S6. Simulated global energy minimum states of a range of giant magnetofossil particles reconstructed from scanning transmission electron microscopy (STEM) tomography (a-t; Pei et al. 2025), and hypothesized spindle-shaped particles from two-dimensional transmission electron microscopy (TEM) and scanning electron microscopy (SEM) (u-x; Xue et al., 2022) (Table S1). Arrows are coloured by the normalized magnetization in the direction perpendicular to the page. Vortex cores are coloured orange.

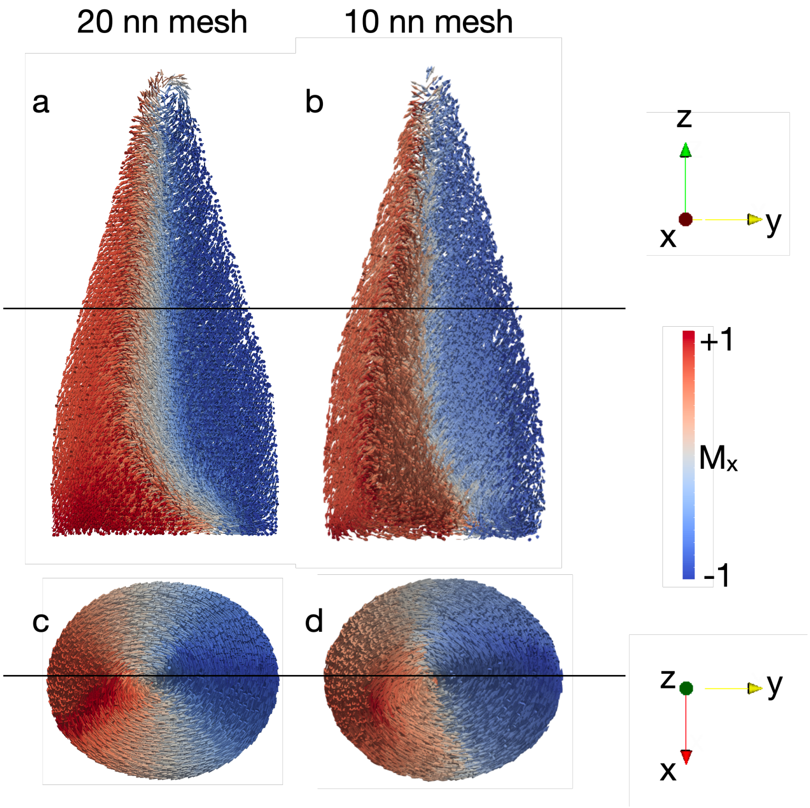

Figure S7. Comparison of micromagnetic simulations performed using a 20 nm mesh (a, c) versus a 10 nm mesh (b, d). Simulations were performed using the same elliptical cross section model used for Fig. 3 and with the [113] axis parallel to the length of the particle (y-z plane). Colours represent the M_x component of magnetisation in all panels. Black line in a and b shows the clipping plane normal to z used to create the images in c and d. Black line in c and d shows the clipping plane normal to x used to create the images in a and b. Results are identical in terms of general features but there are some small differences in detail. The remanent moments are 1×10^{-14} Am^2 for the 20 nm simulation and 0.68×10^{-14} Am^2 for the 10 nm simulation. Total energy densities are -2911 J/m^3 for the 20 nm simulation and -2457 J/m^3 for the 10 nm simulation.

Table S1. Saturation isothermal remanent magnetization (SIRM) moments calculated in 20 external field directions and the global energy minimum (GEM) remanent moments. Giant bullet (GB), kinked giant bullet (KGB), needle (N), cone spearhead (CSH), no-stalk spearhead (NSSH) and whole spearhead (WSH) particles were reconstructed from scanning transmission electron microscopy (STEM) tomography (Pei et al., 2025). Spindle (SPD) particles are hypothesized three-dimensional geometric model based on two-dimensional transmission electron microscopy (TEM) and scanning electron microscopy (SEM) (Xue et al., 2022). SD: single-domain; SV: single-vortex; MV: multi-vortex. See Fig. S6 for GEM states.

Particle	Type	Long axis	Length (nm)	Width (nm)	Height (nm)	2L/(W+H)	Average SIRM moment ($10^{-16}Am^2$)	GEM domain state	GEM moment ($10^{-16}Am^2$)
GB-1	Giant bullet	[111]	976.75	372.0	325.32	2.8	12.5	SV	27.81
GB-2	Giant bullet	[111]	949.99	290.14	261.41	3.44	14.95	SV	29.71
GB-3	Giant bullet	[111]	989.97	443.29	374.78	2.42	12.34	SV	24.45
GB-4	Giant bullet	[111]	944.09	281.66	277.14	3.38	14.57	SV	27.88
GB-5	Giant bullet	[111]	803.52	340.23	313.11	2.46	9.53	SV	14.23
GB-6	Giant bullet	[111]	846.99	342.52	322.8	2.55	10.08	SV	19.69
GB-7	Giant bullet	[111]	556.66	282.33	212.23	2.25	3.96	SV	3.1
KGB-1	Kinked giant bullet	[111]	1169.11	262.18	218.4	4.87	84.34	～SD	166.65
KGB-2	Kinked giant bullet	[111]	1162.25	352.81	225.85	4.02	108.2	～SD	217.5
N-1	Needle	[111]	550.53	125.27	104.83	4.79	10.89	SD	21.48
N-2	Needle	[111]	852.85	150.5	87.78	7.16	15.56	SD	30.78
N-3	Needle	[111]	862.41	140.35	103.88	7.06	18.64	SD	36.89
N-4	Needle	[111]	942.96	100.71	100.69	9.36	13.41	SD	26.58
N-5	Needle	[111]	745.79	105.45	104.29	7.11	12.52	SD	24.77
CSH-1	Cone spearhead	[311]	750.13	586.67	571.27	1.3	8.54	～SV	8.88
WSH-1	Whole spearhead	[110]	1503.06	879.18	608.96	2.02	11.06	SV-MV	25.22
NSSH-1	No-stalk Spearhead	[311]	1790.22	1040.78	798.24	1.95	49.11	SV-MV	94.96
WSH-2	Whole spearhead	[110]	2060.48	1069.1	773.81	2.24	47.37	SV-MV	81.86
CSH-2	Cone spearhead	[311]	623.41	458.85	341.5	1.56	5.72	SV	13.40
WSH-3	Whole spearhead	[110]	2427.06	1033.47	728.13	2.76	52.77	SV-MV	75.71
SPD-1	Spindle	[110]	908.16	182.15	182.15	4.99	-	SV	40.99
SPD-2	Spindle	[110]	1533.3	378.02	378.02	4.06	-	SV	145.52
SPD-3	Spindle	[110]	1918.1	380.5	380.5	5.04	-	SV	231.1
SPD-4	Spindle	[110]	2530.4	409.06	409.06	6.19	-	SV	430.28

Table S2. Micromagnetic simulated susceptibility of six conventional magnetotactic bacteria chain models from Pei et al. (2023).

MTB type	Ref	Crystal form	Susceptibility (10^{-16}Am^2/T)
MSR-1	Uebe & Schüler, 2016	Cuboctahedron	2.78
AMB-1	Li et al., 2020	Cuboctahedron	14.66
SHHR-1	Li et al., 2020	Prism	1.20
XJHC-1	Liu et al., 2021	Prism	8.68
WYHR-1	Li et al., 2019	Bullet	5.07
Rod	Amor et al., 2020	Bullet	17.69

Supplementary References

Amor, M., Mathon, F.P., Monteil, C.L., Busigny, V. & Lefevre, C.T. 2020 Iron-biomineralizing organelle in magnetotactic bacteria: function, synthesis and preservation in ancient rock samples. *Environ. Microbiol.* 22, 3611-3632.

Li, J., Menguy, N., Roberts, A.P., Gu, L., Leroy, E., Bourgon, J., Yang, X.a., Zhao, X., Liu, P., Changela, H.G., et al. 2020 Bullet-shaped magnetite biomineralization within a Magnetotactic Deltaproteobacterium: Implications for magnetofossil identification. *J. Geophys. Res.: Biogeosci.* 125

Liu, P.Y., Liu, Y., Zhao, X., Roberts, A.P., Zhang, H., Zheng, Y., Wang, F.X., Wang, L.S., Menguy, N., Pan, Y.X., et al. 2021 Diverse phylogeny and morphology of magnetite biomineralized by magnetotactic cocci. *Environ. Microbiol.* 23, 1115-1129.

Li, J.H., Zhang, H., Liu, P.Y., Menguy, N.O.L., Roberts, A.P., Chen, H.T., Wang, Y.Z. & Pan, Y.X. 2019 Phylogenetic and structural identification of a novel Magnetotactic Deltaproteobacteria strain, WYHR-1, from a freshwater lake. *Appl. Environ. Microbiol.* 85.

Pei, Z., Chang, L., Bai, F., & Harrison, R. J. (2023). Micromagnetic calculation of the magnetite magnetosomal morphology control of magnetism in magnetotactic bacteria. Journal of the Royal Society Interface, 20(206)

Pei, Z., Ringe, E., Chang, L., Harrison, R.J., Xue, P., Williams, W., 2025. Three-dimensional structure, crystallography, and magnetism of giant magnetofossils. Commun Earth Environ 6, 410.

Uebe, R. & Schüler, D. 2016 Magnetosome biogenesis in magnetotactic bacteria. *Nat. Rev. Microbiol.* 14, 621-637.

Winklhofer, M., Kirschvink, J.L., 2010. A quantitative assessment of torque-transducer models for magnetoreception. Journal of The Royal Society Interface 7, S273–S289.

Xue, P., Chang, L., Pei, Z., Harrison, R.J., 2022. Discovery of giant magnetofossils within and outside of the Palaeocene-Eocene Thermal Maximum in the North Atlantic. Earth and Planetary Science Letters 584, 117417.
