# Supplementary material for: Magnetic vector tomography reveals giant magnetofossils are optimised for magnetointensity reception
Source: Commun Earth Environ. 2025 Oct 20;6(1):810. doi: 10.1038/s43247-025-02721-3 (PMC12537488; doi:10.1038/s43247-025-02721-3)
Supplement: Supplementary file 3 — Description of Additional Supplementary Files [file 43247_2025_2721_MOESM3_ESM.pdf]

Description of Additional Supplementary Files

Supplemental Movie S1. 3D reconstruction movie.

Supplemental Movie S2. 3D longitudinal slices movie.

Supplemental Movie S3. Micromagnetic simulations performed with an elliptical cross section (r_x = 0.55 µm; r_y = 0.4675 µm), [113] parallel to *z* and [111] (the magnetocrystalline easy axis) in the *x*-*z* plane (yellow and green axes, respectively). Surface spins are coloured according to the component of magnetisation normal to the plane of the diagram (*y*). The vortex cores are coloured according to M_z. A series of 50 local energy minimum (LEM) states are shown, generated by taking random starting configurations and performing energy minimisation in zero field. Repeated energy minimisation from a random starting configuration produces multiple local energy minimum (LEM) states, most commonly double/multi-vortex states containing multiple BPs. Only a small subset of LEMs display features that closely resemble the observed 3D reconstruction. Only 2 out of the 50 states (states 48 and 50) correspond to the global energy minimum (GEM), which consists of a single vortex with no Bloch points. The snapshot above shows a double vortex state.

Supplemental Movie S4. Micromagnetic simulation of tip-down growth of a giant magnetofossil. Each stage of growth involved adding a 25 nm thick frustrum with randomly initialised spins to the pre-existing model and then energy minimising. Central slice is coloured according to the M_z component of magnetisation.

Supplemental Movie S5. Micromagnetic simulation of base-up growth of a giant magnetofossil. Each stage of growth involved adding a 25 nm thick frustrum with randomly initialised spins to the pre-existing model and then energy minimising. Central slice is coloured according to the M_z component of magnetisation.

Supplemental Movie S6. Nudged elastic band (NEB) simulation of Bloch point nucleation and propagation performed with a circular cross section (r_x = 0.55 µm), [113] parallel to *z* and [111] (the magnetocrystalline easy axis) in the *x*-*z* plane (yellow and green axes, respectively). NEB minimum action path was calculated using 100 steps, with the configurations at the ends of the path corresponding to equivalent GEM states with identical helicity but oppositive vortex core magnetization. The Bloch point nucleates preferentially at the tip, whereas destruction of the Bloch point occurs via a different (much higher energy) mechanism at the base (see Fig. S4).
